# Ontario Veterinary College

**Published:** 1897-05

**Authors:** 


### ONTARIO VETERINARY COLLEGE.

The exercises at the closing of the session of 1896–'97 took place on March 26th. The Principal, Prof. A. Smith, F.R.C.V.S., in the chair; beside him on the platform were the members of the Faculty and a board of examiners, most of whom are prominent veterinary surgeons who are practising their profession in Canada and the United States.

Prof. Smith opened the meeting with a short but eloquent address. He congratulated the successful students on passing the searching ordeal of a final examination. They were now about to engage in the "battle of life" by putting the knowledge they had been acquiring by study into actual practice, and he most heartily wished them every success in their chosen profession.

Mayor Lloyd gave the recent graduates some excellent advice, founded on many years' experience, as to their conduct and deportment with the public at large, urging the necessity for aiming at a high standard; of honorable dealing with the public at large, as well as to still keep pressing onward in the race for knowledge, as only by those means could future success be assured.

Prof. J. H. Reed, of the Guelph Agricultural College, also gave a short address; among other matters, he particularly mentioned that a proper standard of professional ethics and etiquette should be strictly observed in our dealings with brother practitioners; that we should each and all strongly disapprove of deprecatory remarks directed against our neighboring brethren, and by no means to class them as opponents, but as all pressing forward for the same goal

—the advancement of our own knowledge and the advancement of the profession at large.

The addresses were all warmly applauded by the students and graduates.

The degree of V.S. was then conferred on the following gentlemen:

E. L. Bertram, Strausstown, Pa.; George C. Bowen, Newark, N. J.; Wallace P. B. Brennan, Parkhurst, Quebec; Frank W. Bryant, Sunderland, Ontario; Clinton H. Bugbee, Keene, N. H.; Henry Buss, Deansville, N. Y.

Wallace Caister, Tavistock, Ontario; George R. Caldwell, Ashton, R. I.; John B. Campbell Grandin, N. Dak.; Herbert B. Chaney, Akron, Ohio; Robert C. Cliff, Hamilton, Ontario; T. J. Cooper, Lockport, N. Y.; W. R. Cross, Beeton, Ont.; Clarence W. Clark, New Britain, Conn.

Wilson S. Decker, Scranton, Pa.; John Dodd, Glasgow, Scotland; James A. Durkin, London, Ont.

Thomas A. Ewart, Wyoming, Ont.; W. H. Erwin, Mason, Mich.

Robert J. Farley, Toronto, Ont.; Andrew L. Fosse, Deerfield, Wis.

George J. Grange, Guelph, Ont.

George Hilton, Winnipeg, Manitoba; Fred. R. Hodgson, Forest, Ont.; Fred. A. Hoskin, Forest, Ont.; Charles S. Horne, Walnut Grove, Miss.

John N. Johnston, Petersborough, Ont.

Fred. Kemmer, Connersville, Ind.; Newton A. Kippen, Tiverton, Ont.

John J. Linden, Canandaigua, N. Y.

W. Frederick McElroy, Bewdley, Ont.; William O. McHugh, Bridgeport, Ohio; G. A. McKenzie, Deloraine, Manitoba; D. J. McKillop, Bristol, Quebec; John A. McKinnon, Sonya, Ont.; Alfred A. McLachlan, St. Johns, Newfoundland; W. McCorkindale, Toronto, Ont.; C. S. Macdonald, Toronto; Ont.; Rudolph J. Marshall, Hoosick Falls, N. Y.; Wilfred J. Meloche, Ogdensburg, N. Y.; Walter D. Monk, South March, Ont.

Alfred J. Nelson, Stoughton, Wis.; Isaac S. Norton, Marshall, Minn.

Robert D. Orr, Baden, Ont.; Thomas M. Owen, Gaithersburg, Maryland.

Jesse H. Pierce. Smithfield, Pa.; Charles A. Prouty, Putney, Vermont; George S. Price, Fenwick, Ont.; Howard F. Pegan, Cochranton, Pa.

W. W. Richards, San Diego, Cal.; J. R. F. Rochester, Denton, Maryland; G. Vernon Rowcroft, Birtle, Manitoba.

Andrew Seim, Ayton, Ont.; D. K. Smith, Toronto, Ont.; Lyndon S. Smith, Cornish, N. H.; George P. Statter, Sioux City, Iowa; Jasper J. Smart, Sherborn, Mass.

Charles E. Taylor, Sawyerville, Quebec; A. E. Tweedie, Ivanhoe, Ont.

J. Eugene Underhill, Middletown, Conn.

Charles E. Virtue, Mt. Gilead, Ohio.

William H. Wheeler, Stamford, N. Y.; John W. Will, Petrolia, Ont.; J. A. H. Winsloe, Buffalo, N. D.

The prize winners were as follows:

*Disease and Treatment.* Seniors: First prize, silver medal, G. Hilton; second prize, H. B. Chaney; third prize, E. L. Bertram, R. C. Cliff, and W. W. Richards. Honors: F. W. Bryant, G. R. Caldwell, W. J. Meloche, A. A. McLachlan, T. M. Owen, J. R. F. Rochester, G. V. Rowcroft, A. Seim, L. S. Smith, W. H. Wheeler, and J. A. H. Winsloe.

*Materia Medica.* Seniors: First prize, George Hilton; second prize, Robert C. Cliff; third prize, Wilfred J. Meloche. Honors: Frank W. Bryant, Clinton H. Bugbee, Herbert B. Chaney, Wallace Caister, George B. Caldwell, and W. W. Richards.

*Chemistry.* Seniors: First prize, R. C. Cliff; second prize, G. Hilton and R. J. Farley; third prize, H. Buss. Honors: C. H. Bugbee, G. C. Bowen, G. R. Caldwell, A. Ewarts, N. Kippen, W. D. Monk, W. J. Meloche, W. O. McHugh, J. R. Rochester, G. V. Rowcroft, A. E. Tweedie, and W. H. Wheeler.

*Morbid Anatomy.* Seniors: First prize, G. Hilton; second prize, T. J. Cooper. Honors: E. L. Bertram, H. Buss, C. H. Bugbee, G. R. Caldwell, R. C. Cliff, H. Chaney, H. Kippen, G. V. Rowcroft, and W. H. Wheeler.

*Anatomy.* Seniors: First prize, silver medal, R. C. Cliff; second prize, G. Hilton; third prize, W. O. McHugh. Honors: E. L. Bertram, G. C. Bowen, F. W. Bryant, C. H. Bugbee, H. Buss, W. Caister, G. R. Caldwell, H. Chaney, W. Cross, T. J. Cooper, W. S. Decker, J. Dodd, R. J. Farley, F. Hodgson, F. A. Hoskin, J. N. Johnson, N. Kippen, W. F. McElroy, G. A. McKenzie, W. J. Meloche, R. D. Orr, T. M. Owen, G. V. Rowcroft, L. S. Smith, G. P. Statter, C. E. Virtue, W. H. Wheeler, and J. A. H. Winsloe.

*Entozoa.* Seniors: First prize, C. H. Bugbee. Honors: E. L. Bertram, H. Buss, G. R. Caldwell, T. J. Cooper, R. J. Farley, G. Hilton, A. J. McKillop, W. J. Meloche, W. O. McHugh, J. R. T. Rochester, W. W. Richards, and W. H. Wheeler.

*Dissected Specimens.* Seniors: Gold medal, given by the Toronto Industrial Exhibition Association, C. S. Macdonald.

*Physiology.* Seniors: First prize, George Hilton; second prize, W. O. McHugh; third prize, E. L. Bertram. Honors: H. Buss, C. H. Bugbee, Frank W. Bryant, Robert C. Cliff, T. J. Cooper, and W. W. Richards.

*Best General Examination.* Gold medal, given by the Ontario Veterinary Association, R. C. Cliff. Honors: H. Chaney, G. Hilton, and W. J. Meloche.

PRIMARY EXAMINATION.—*Anatomy.* Seniors: J. O. Henry, L. Pauquette, R. P. Scandrett, Winfield Wallace.

*Materia Medica.* H. Paine.

*Disease and Treatment.* Juniors: First prize, C. W. Fisher; second prize, J. S. Pollard; third prize, E. R. Stockwell and W. H. Shaw. Honors: W. L. Adams, L. Bailey, S. Caldbick, W. R. Clark, W. H. Corey, R. B. Coutts, W. G. Huyett, C. H. Jewell, A. Jordan, T. Lambrechts, E. H. Hawley, D. McKenzie, R. Macdonald, J. W. Rutledge, A. Sorensen, H. W. Stedman, T. Sims, Samuel Shepard Treadwell, A. G. Vantine, and A. C. Walker.

*Anatomy.* Juniors: Silver medal, C. W. Fisher; second prize, T. Lambrechts; third prize, J. S. Pollard. Honors: John Black, W. D. Brand, L. Bailey, S. Caldbick, R. B. Coutts, L. T. Dunn, G. W. Higginson, W. Huyett, C. I. Irons, C. H. Jewell, G. H. Lawley, J. A. McDonald, A. D. McLachlan, R. McDonald, D. McKenzie, J. W. Rutledge, John Short, W. H. Shaw, E. R. Stockwell, T. Sims, C. Stevenson, H. W. Steadman, S. S. Treadwell, A. Walker, and S. J. Waterman.

*Physiology.* Juniors: First prize, C. W. Fisher; second prize, C. H. Jewell; third prize, T. Rowland. Honors: L. Bailey, E. H. Lawley, T. Lambrechts, D. McKenzie, J. S. Pollard, and T. Sims.

*Chemistry.* Juniors: First prize, C. W. Fisher; second prize, T. Rowland. Honors: W. D. Brand, L. Bailey, G. R. Cranston, E. C. Elwes, G. F. Irons, A. Jordan, E. H. Lawley, T. Lambrechts, Milton McClellan, D. McKenzie, H. Moore, J. S. Pollard, J. A. Raleigh, T. Sims, E. R. Stockwell, and S. J. Waterman.

*Histology and Microscopy.* Juniors: First prize, C. W. Fisher; second prize, G. F. Irons; third prize, S. S. Treadwell. Honors: L. Bailey, W. D. Brand, A. Jordan, E. H. Lawley, T. Lambrechts, R. Macdonald, D. McKenzie, and T. Rowland.

In closing, Mr. C. S. Macdonald, President of the graduating class, presented Prof. Smith with a magnificent picture of the class, to which the Principal responded.

In the evening a banquet was given by the Ontario Veterinary Association, presided over by Prof. Smith.

